# Acute Lymphoblastic Leukemia Presenting Solely as Low Back Pain

**DOI:** 10.5811/cpcem.2019.1.40699

**Published:** 2019-01-29

**Authors:** Joshua Goodwin, Bijon Das

**Affiliations:** Dalhousie University, Department of Emergency Medicine, Halifax, Nova Scotia

## Abstract

A 23-year-old man with acute lymphoblastic leukemia presented to the emergency department without any history of constitutional symptoms (fatigue, anorexia, or weight loss), dyspnea, bruising, or bleeding. Presentation of acute leukemia solely as musculoskeletal pathology is common in pediatric populations but rare among adult patients. Recognizing this presentation of acute leukemia in adult patients could help prevent delayed diagnoses.

## INTRODUCTION

Acute lymphoblastic leukemia (ALL) is the second most common acute leukemia in the United States, with more than 6,500 new cases annually.^1^ The majority (80%) of ALL cases are diagnosed in children.^1^ In Canada, an estimated 5,900 patients were diagnosed with leukemia in 2016, with rates of 24% for acute myeloid leukemia (AML), and 5% for ALL.^2^ Both AML and ALL typically present with constitutional symptoms such as fatigue, anorexia, weight loss, and sequelae of bone marrow failure, which include increased bleeding, easy bruising, infection, and dyspnea.^1^,^3^ Patients with ALL also commonly present with fever, night sweats, lymphadenopathy, splenomegaly, or hepatomegaly; in some cases, central nervous system involvement is seen.^2^ Musculoskeletal symptoms are uncommon in cases of acute leukemia in adults.^4^–^6^ In some rare cases, acute leukemia can present solely as bone pain, although this presentation is more commonly seen in children.^7^,^8^ This case report demonstrates the importance of keeping leukemia on the differential for patients presenting solely with lower back pain in order to prevent a delayed diagnosis.

## CASE REPORT

A 23-year-old male presented to the emergency department (ED) with pain in his lower back radiating down his left leg. He had awoken from sleep five days prior with lower back pain radiating into both legs. He went to a hospital in another city where he was prescribed morphine, cyclobenzaprine, and naproxen. His pain was initially controlled enough that he could return to work while taking naproxen. The pain continued to progressively worsen and had begun to cause difficulty walking. He went to a family physician who suspected sciatic nerve pain and sent him to the ED for imaging of his spine.

On presentation to the ED, his pain was 7 to 8.5 out of 10. There was no history of trauma, and he had no bowel incontinence, urinary retention, or saddle anesthesia. He had no recent fevers, chills, or weight loss. His appetite was decreased due to his pain, and he had not had a bowel movement in three days. He had no significant past medical history and usually took no medications. He had consumed seven to nine alcoholic drinks and used cocaine the night before the pain began.

On examination, his temperature was 37.3°C, his heart rate was 96 beats per minute, his respiratory rate was 16 breaths per minute, his blood pressure was 124/60 millimeters of mercury, and his oxygen saturation was 100%. His abdomen was soft and non-tender. Testicular and rectal exams were normal. Palpation of the left sacroiliac joint revealed exquisite tenderness, identifying the more precise location of the lower back pain. Neurologic exam revealed normal tone, strength, and coordination in all extremities. Radiographs of the pelvis and sacroiliac joints were normal.

On laboratory workup, white cell count was 6.89×10^9^/liter (L) (normal range, 4.5–11), serum hemoglobin was 100 grams (g)/L (normal range, 140–180), platelet count was 143×10^9^/L (normal range, 150–350), and C-reactive protein (CRP) was 227 milligrams/L (normal range, <8). Peripheral blood smear showed increased polychromasia, some poikilocytosis with occasional teardrop cells, mature neutrophils, roughly 20% circulating blasts, and rare giant platelets, all consistent with acute leukemia. In the ED he was administered two milligrams (mg) of hydromorphone and 600 mg of ibuprofen orally. He was later admitted to the hematology service. He was diagnosed with precursor B-cell ALL and initiated on the Dana-Farber chemotherapy protocol. Two years post-diagnosis, his leukemia was in remission and he had recently completed his final cycle of chemotherapy.

## DISCUSSION

This otherwise-healthy, 23-year-old male patient was diagnosed with leukemia after presenting solely with lower back pain. Initial differential diagnoses considered for this patient by the attending emergency physician (EP) included cauda equina syndrome, sciatic nerve pathology, trauma to the lower back, spinal metastases, rheumatological disorders, and infection. Given that the patient stated he had used alcohol and cocaine the night before the pain began, rhabdomyolysis was also considered. In an otherwise-healthy young patient with recent recreational drug use on history, one might also keep drug-seeking behaviour on the differential.

Physical examination and history ruled out the possibility of a cauda equina syndrome, trauma, or a previously diagnosed malignancy. The exquisite tenderness of the patient’s sacroiliac joint on palpation suggested pathology of the sacroiliac joint or associated bones rather than sciatic nerve pathology. This was a distinguishing feature of this patient’s presentation, likely precipitated by cellular proliferation in bone of the left sacroiliac joint.

The possibility of bone marrow proliferation from leukemia being the cause of the lower back pain was considered by the attending EP once the patient’s peripheral blood smear was found to contain circulating blasts. Acute leukemia is not known to typically present as pain in the large joints such as the sacroiliac,^4^ and in this case none of the typical signs of leukemia such as fatigue, bruising, or easy bleeding were present to aid in a clinical diagnosis.^1^,^2^ To our knowledge, no cases of leukemia presenting solely with back pain are present in the emergency medicine literature. There have been cases where adult patients’ acute leukemia has presented as pain in joints involving the back, but these were accompanied by pain in the long bones that is more typical of leukemia.^4^,^5^ Results of the peripheral blood smear aside, an undiagnosed rheumatological disorder might also have been considered as a potential etiology. If blood tests had not been ordered in the case of this healthy young patient, his diagnosis could have been missed while his leukemia continued to progress undetected.

An otherwise-healthy young patient presenting to the ED with a sole complaint of lower back pain in the absence of trauma could raise suspicion for leukemia and prompt the EP to order a complete blood count and peripheral blood smear. Both a strong opioid and a non-steroidal anti-inflammatory (or acetaminophen) are suggested for cancer pain rated by the patient to be greater than 6 out of 10 on a numerical rating scale,^9^ and both hydromorphone and ibuprofen were required for effective pain management in this case.

**CPC-EM Capsule**

What do we already know about this clinical entity?

*Leukemia can present solely as musculoskeletal pain, but this presentation is typically seen in children*.

What makes this presentation of disease reportable?

*There are no previously reported cases of a leukemia presenting solely as low back pain*.

What is the major learning point?

*Leukemia could be considered in the differential diagnosis of new-onset, low back pain in an otherwise-healthy young adult with no history of physical trauma*.

How might this improve emergency medicine practice?

*Considering leukemia in the differential diagnosis for these patients could prevent a missed diagnosis of leukemia*.

## CONCLUSION

In this case, acute leukemia was diagnosed in a patient presenting with a sole clinical feature of lower back pain. This diagnosis would typically be much lower on the differential for a purely musculoskeletal complaint. To prevent delay in diagnosis and appropriate treatment, it is important to recognize leukemia as a potential cause of severe lower back pain in an otherwise-healthy patient presenting to the ED.

## References

[1] Terwilliger T, Abdul-Hay M (2017). Acute lymphoblastic leukemia: a comprehensive review and 2017 update. Blood Cancer J.

[2] Hodgson CS Blood Cancer in Canada Facts & Stats 2016.

[3] De Kouchkovsky I, Abdul-Hay M (2016). Acute myeloid leukemia: a comprehensive review and 2016 update. Blood Cancer J.

[4] Sakata H, Nakao A, Matsuda K (2014). Acute leukemia presenting as bone pain with normal white blood cell count. Acute Med Surg.

[5] Mohanty PK, Patel DK, Majhi CD (2004). Aleukemic acute lymphoblastic leukemia in adult presenting with rheumatic manifestations and osteolytic vertebral lesions. J Indian Rheumatol Assoc.

[6] Shapiro FD, Leval LD (2000). Case records of the Massachusetts General Hospital (Case 32 – 2000); A boy with vertebral compression fracture. N Eng J Med.

[7] Brix N, Rosthøj S, Herlin T (2015). Arthritis as presenting manifestation of acute lymphoblastic leukaemia in children. Arch Dis Child.

[8] Chell J, Fernandes JA, Bell MJ (2001). The orthopaedic presentation of acute leukaemia in childhood. Ann R Coll Surg Engl.

[9] Ripamonti CI, Bandieri E, Roila F (2011). Management of cancer pain: ESMO clinical practice guidelines. Ann Oncol.

